# Baicalein and Αlpha-Tocopherol Inhibit Toll-like Receptor Pathways in Cisplatin-Induced Nephrotoxicity

**DOI:** 10.3390/molecules27072179

**Published:** 2022-03-28

**Authors:** Amira Awadalla, Mohamed R. Mahdi, Mohamed H. Zahran, Ahmed Abdelbaset-Ismail, Mohamed El-Dosoky, Amr Negm

**Affiliations:** 1 Center of Excellence for Genome and Cancer Research, Urology and Nephrology Center, Mansoura University, Mansoura 35516, Egypt; a.lahlouba@hotmail.com; 2 Department of Human Anatomy and Embryology, Faculty of Medicine, Mansoura University, Mansoura 35516, Egypt; m_refaat@mans.edu.eg; 3 Urology Department, Urology and Nephrology Center, Mansoura University, Mansoura 35516, Egypt; zahranmha822@gmail.com; 4 Department of Surgery, Anesthesiology and Radiology, Faculty of Veterinary Medicine, Zagazig University, Zagazig 44519, Egypt; a4azzazy@yahoo.com; 5 Department of Neuroscience Technology, College of Applied Medical Science in Jubail, Imam Abdulrahman Bin Faisal University, Jubail 34221, Saudi Arabia; mesalama@iau.edu.sa; 6 Department of Chemistry, College of Science, King Faisal University, Al-Ahsa 31982, Saudi Arabia; 7 Biochemistry Division, Chemistry Department, Faculty of Science, Mansoura University, Mansoura 35516, Egypt

**Keywords:** nephrotoxicity, cisplatin, baicalein, alpha-tocopherol, antioxidant

## Abstract

Cisplatin (CP) is a conventional chemotherapeutic agent with serious adverse effects. Its toxicity was linked to the stimulation of oxidative stress and inflammation. As a result, this study explored the protective effect of baicalein and alpha-tocopherol in nephrotoxicity induced by cisplatin. Until receiving an intraperitoneal injection of CP (3 mg/kg BW), rats were given baicalein orally 100 mg/kg for seven days or/and a single intraperitoneal injection of α-tocopherol 250 mg/kg. Renal function was tested to explore whether baicalein and α-tocopherol have any beneficial effects; blood urea nitrogen (BUN), serum creatinine, malondialdehyde (MDA) content, antioxidant activity biomarkers and histopathology of renal tissue, oxidative stress biomarkers, inflammatory response markers, and histopathological features of kidney architecture were measured. Cisplatin treatment resulted in extreme renal failure, as measured by high serum creatinine and BUN levels and severe renal changes. Cisplatin therapy resulted in increased lipid peroxidation and decreased glutathione and superoxide dismutase levels, reflecting oxidative stress. Upon treatment with α-tocopherol, baicalein, and combined therapy, there was augmentation in the antioxidant status as well as a reduction in IL-6, NF-κB, TNF, TLR2, and TLR4 and a significant increase in Keap-1 and NRF-2. The combined treatment was the most effective and the nearest to the normal status. These findings suggest that baicalein and α-tocopherol may be useful in preventing cisplatin-induced nephrotoxicity.

## 1. Introduction

Cisplatin is used to treat a variety of solid tumors [1]. Regrettably, the therapeutic use of cisplatin is restricted because of its side effects on different body organs, especially nephrotoxicity [2]. It is associated with a 3% decrease in glomerular filtration rate with subsequent increment in serum creatinine and BUN, days after treatment initiation [3].

Until now, the mechanism of cisplatin-induced nephrotoxicity (CIN) is incompletely clear. Recent studies attributed CIN to the oxidative process and accumulation of oxidative stress molecules [4] and renal cell apoptosis [5]. Cisplatin was identified to increase reactive oxygen species (ROS) by enhancing cellular macromolecules oxidation. Furthermore, it decreased the efficiency of antioxidant enzymes, which generate cellular oxidative stress [4].

ROS produce a massive amount of the inflammatory cytokine “tumor necrosis factor-α” (TNF-*α*), which induces the inflammatory process through cytokine production [6]. Toll-like receptors (TLRs) stimulate MAPK and NF-κB pathways, which trigger and stimulate the release of inflammatory mediators [7]. Prevention and treatment of CIN can be achieved by reversing the inflammatory response by reducing inflammatory mediators and controlling the oxidative stress activity. Nrf2 transcription factor is a critical factor in induction of inflammatory process. Its activation is needed to regulate the cellular oxidative stress and inhibit CIN, while Nrf2 absence aggravates it [8]. Therefore, finding a compound with the potential to reduce inflammation and oxidative stress by activating Nrf2 is highly needed.

**Figure 1:**
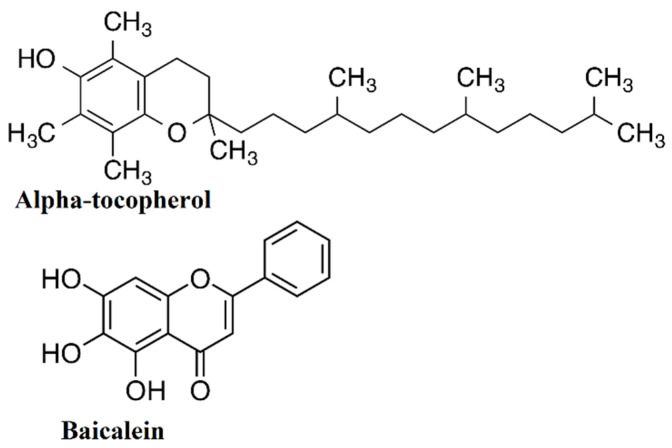
Chemical structure of α-tocopherol and baicalein.

Baicalein (7-glucuronic acid,5,6-dihydroxyflavone) (Figure 1) is a root extract from *Scutellaria baicalensis* plant, which possess antiinflammatory, antimacrobiotic, and immunoregulatory activities [9]. Previous studies showed that baicalein decreases DNA damage and apoptosis [10]. It reduced renal fibrosis by downregulating the MAPK and NF-kB pathways in a rat model [11]. Furthermore, studies showed that baicalein pretreatment could significantly ameliorate kidney damage, by lowering proinflammatory cytokines, and apoptosis, through inhibiting the TLR4 pathway [12], as well as protecting the kidney against colistin-induced nephrotoxicity via enhancing the Nrf2 pathway, a master transcription factor controlling the oxidative stress process [13].

Vitamin E has eight isoforms; α-tocopherol is one of them and is the most effective fat-soluble natural antioxidant. It has a promising protective and therapeutic effect on heart disease, Alzheimer’s, and cancer [14,15]. α-Tocopherol reduced the oxidative stress parameters in L-thyroxine-induced stress [16]. Combined antioxidant vitamins E and C and a natural antioxidant extract elevated antioxidant enzyme activity and total superoxide scavenger activity (TSSA) with a significant decrease in malondialdehyde (MDA) level [17]. Furthermore, Feng et al. (2010) demonstrated that besides the direct antioxidant activities of α-tocopherol, “such as iron chelation and free radical scavenging and, another mechanism of the protective effects of α-tocopherol proceeds by induction of the phase II enzyme response via the transcription factor Nrf2” [18].

These findings suggest that combined baicalein and α-tocopherol could be an effective renoprotective treatment of CIN regarding their antioxidant capability. Thus, the current study aimed to assess the value of inhibition of toll-like receptors and oxidative stress activity by both compounds on CIN and identify the possible mechanisms.

## 2. Results

### 2.1. Effect of α-Tocopherol and Baicalein Administration on Renal Function in Control and Treated Groups

**Figure 2:**
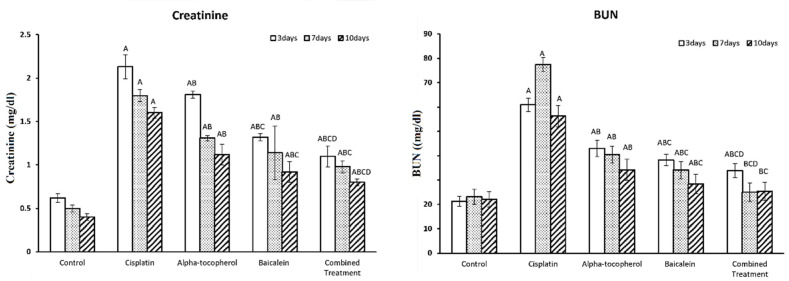
Impact of α-tocopherol and baicalein administration on kidney function. All data are expressed as mean ± SEM. Significant vs. control group refers to A, while significant vs. (cisplatin, -tocopherol, and baicalein) treatment groups refers to (B, C, D), respectively.

Figure 2 shows the assessment of the biochemical parameters of renal function at different periods. There was a significant increase in both creatinine and BUN upon cisplatin administration. Upon treatment with α-tocopherol, baicalein, and combined treatment, a significant reduction in both creatinine and BUN was observed.

The decrease was seen gradually, and the intensity increased with an increase in the period of the treatment. The improving effect of baicalein was shown to be greater than that of α-tocopherol, while the combined treatment was the most effective and the nearest to the normal status.

### 2.2. Effect of α-Tocopherol and Baicalein Administration on SOD, GSH, and MDA Levels in Control and Treated Groups

**Figure 3:**
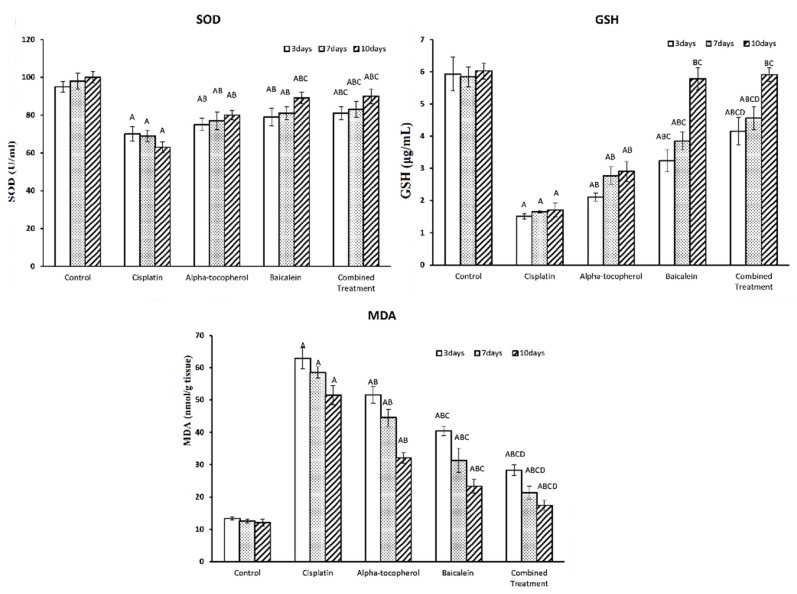
Effect of α-tocopherol and baicalein administration on SOD, GSH, and MDA levels in control and cisplatin-treated groups. All data are expressed as mean ± SEM. Significant vs. control group refers to A, while significant vs. (cisplatin, -tocopherol, and baicalein) treatment groups refers to (B, C, D), respectively.

The assessment of the biochemical parameters of oxidative status, including antioxidant molecules such as SOD and GSH in addition to the lipid peroxidation molecule malondialdehyde (MDA) at different periods of time (3, 7, and 10 days) is shown in Figure 3. There was a significant decline in both SOD and GSH upon administration of cisplatin. Upon treatment with α-tocopherol, baicalein, and combined treatment, SOD and GSH levels increased significantly. The increase was seen gradually and was time-dependent; i.e., the intensity increased with the increase in the period of the treatment. The enhancing of antioxidant status of baicalein was shown to be greater than that of α-tocopherol. The combined treatment was the most effective and the nearest to the normal status. 

Administration of cisplatin significantly increased MDA level. Upon treatment with α-tocopherol, baicalein, and combined treatment, MDA level was significantly decreased. Furthermore, it is obvious that α-tocopherol improved MDA level more than baicalein. The combined treatment was most effective than α-tocopherol or baicalein alone.

### 2.3. Effect of α-Tocopherol and Baicalein Administration on NF-κB, IL-6, and TNF in Control and Treated Groups

**Figure 4:**
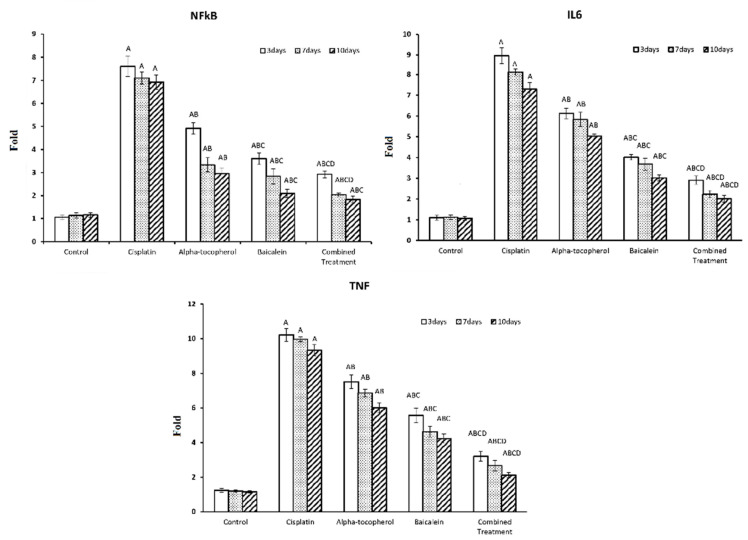
Effect of α-tocopherol and baicalein administration on NF-κB, IL-6, and TNF in control and treated groups. All data are expressed as mean ± SEM. Significant vs. control group refers to A, while significant vs. (cisplatin, -tocopherol, and baicalein) treatment groups refers to (B, C, D), respectively.

Figure 4 shows the assessment of the inflammation modulators such as the transcriptional factor nuclear factor-κB (NF-κB), anti-inflammatory cytokine IL-6, and proinflammatory cytokine TNF at different periods of time (3, 7, and 10 days). There was significant increase in NF-κB, IL-6, and TNF after administration of cisplatin. This reflects the inflammation induction effect of cisplatin. Upon treatment with α-tocopherol, baicalein, and combined treatment, there was a significant decrease in the TNF, IL-6, and NF-κB in nephrotoxicity-induced groups. Baicalein showed better improving action than α-tocopherol. The combined treatment was the most effective and the nearest to the normal status. 

### 2.4. Effect of α-Tocopherol and Baicalein Administration on TLR2 and TLR4 Genes in Control and Treated Groups

**Figure 5:**
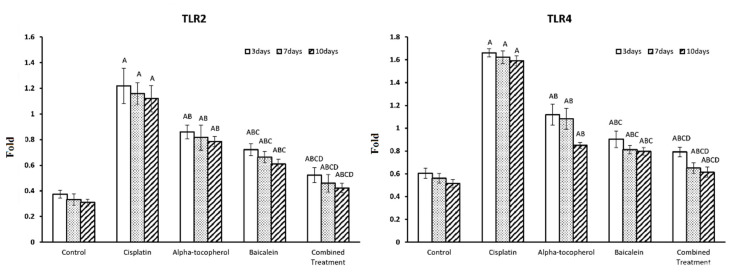
Effect of α-tocopherol and baicalein administration on TLR2 and TLR4 in control and treated groups. All data are expressed as mean ± SEM. Significant vs. control group refers to A, while significant vs. (cisplatin, -tocopherol, and baicalein) treatment groups refers to (B, C, D), respectively.

TLRs are crucial in triggering immunological responses that stimulate cells and induce the release of proinflammatory cytokines, resulting in a greater immunoinflammatory response. Figure 5 shows the assessment of TLR2 and TLR4 at different periods of time (3, 7, and 10 days). TLR2 and TLR4 were significantly increased after the administration of cisplatin. Upon treatment with α-tocopherol, baicalein, and combined treatment, TLR2 and TLR4 were significantly decreased in nephrotoxicity-induced groups. Baicalein showed better improving action than α-tocopherol. The combined treatment was the most effective and the nearest to the normal status.

### 2.5. Effect of α-Tocopherol and Baicalein Administration on Keap-1 and NRF-2 in Control and Treated Groups 

**Figure 6:**
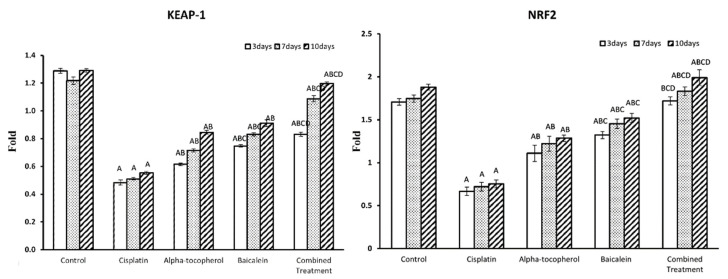
Effect of α-tocopherol and baicalein administration on Keap-1 and NRF-2 in control and treated groups. All data are expressed as mean ± SEM. Significant vs. control group refers to A, while significant vs. (cisplatin, -tocopherol, and baicalein) treatment groups refers to (B, C, D), respectively.

Figure 6 shows the assessment of Keap-1 and NRF-2 at different periods of time (3, 7, and 10 days). There was a significant decrease in Keap-1 and NRF-2 after administration of cisplatin. Upon administration of α-tocopherol, baicalein, and combined treatment, Keap-1 and NRF-2were significantly increased in nephrotoxicity-induced groups. Baicalein showed better improving action than α-tocopherol. The combined treatment was the most effective and the nearest to the normal status.

### 2.6. Effects of α-Tocopherol and Baicalein on Renal Changes

**Figure 7:**
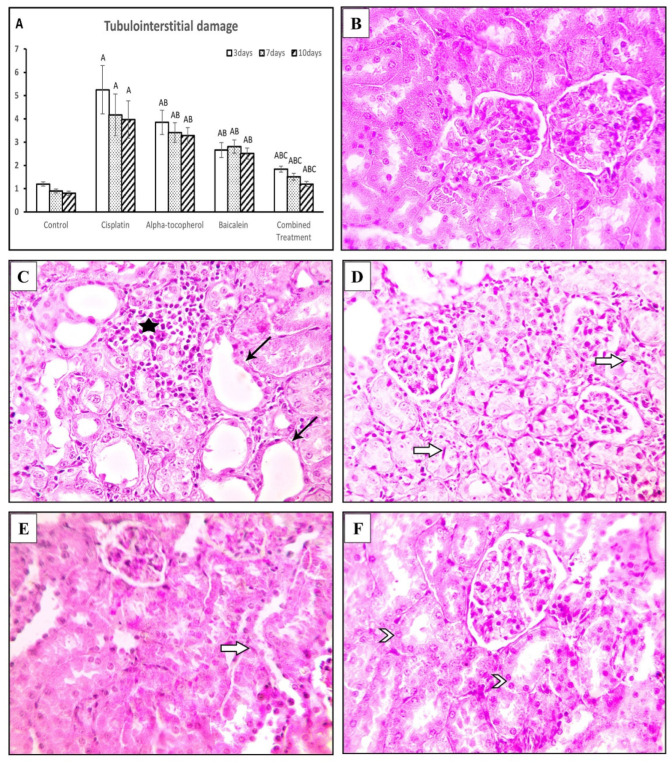
Microscopic pictures of H&E-stained renal sections showing (**A**) tubulointerstitial damage score in treated groups, Significant vs. control group refers to A, while significant vs. (cisplatin, -tocopherol, and baicalein) treatment groups refers to (B, C, D), respectively, (**B**) normal cortex and medulla in the control group, (**C**) leukocytic cell infiltration (star) and dilated tubules (black arrow) in cisplatin group, (**D**) mitotic figure (white arrow) in α-tocopherol group, (**E**) mitotic figure (white arrow) in baicalein group, and (**F**) prominent nuclei (arrowhead) in combined treatment group. High magnification ×: 400 bar 50.

The score of damaged renal tissue was evaluated by morphological analysis of all groups at different times. The histopathological damage score was significantly high in the cisplatin group (*p* < 0.01) and low in α-tocopherol and baicalein groups at the different periods and significantly lower in the combination group than in the α-tocopherol group (*p* < 0.01) (Figure 7A).

Rats sacrificed after 3, 7, and 10 days showed normal cortex and medulla in the control group (Figure 7B). Meanwhile, renal sections from the cisplatin group sacrificed after 3, 7, and 10 days showed the following: marked tubular dilation, degeneration, and necrosis in corticomedullary junction with cast formation and focal leukocytic cell infiltration with few signs of regenerative changes such as tubular dilation with flattened epithelium and tubular cell enlargement with enlarged nuclei in the cortex; tubular dilation with cast formation appeared in the medulla (Figure 7C).

On the other hand, rats treated with α-tocopherol showed moderate tubular dilation in cortex and medulla with low leukocytic cell infiltration in the interstitial tissue and few signs of regenerative changes such as tubular cell enlargement with prominent nuclei (Figure 7D); the baicalein group showed mild tubular dilation and focal leukocytic cell infiltration with increased signs of regeneration such as tubular dilation with flattened epithelium in the cortex (Figure 7E). Meanwhile, renal sections from the combined treatment rat group showed a completely regenerated renal epithelium with normal cortex and medulla (Figure 7F).

### 2.7. Effect of α-Tocopherol and Baicalein on the Immunohistochemical Expression of TLR4, TLR2, and Keap-1

**Figure 8:**
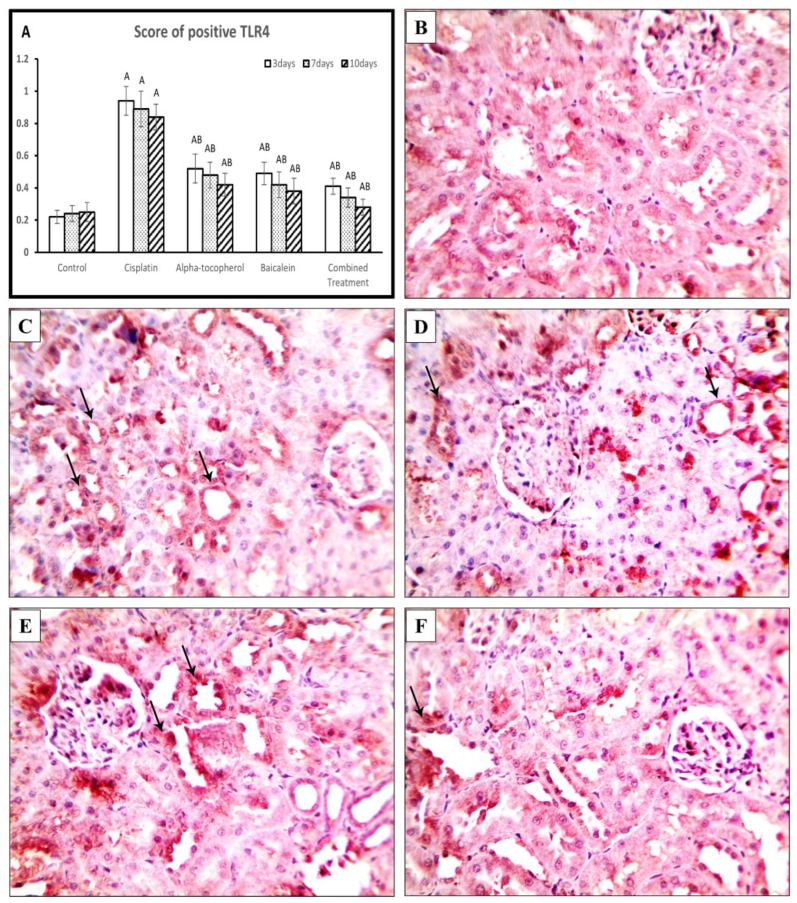
Microscopic pictures of adrenal sections immunostained against TLR4 from rat groups sacrificed after 10 days showing (**A**) positive cell score Significant vs. control group refers to A, while significant vs. (cisplatin, -tocopherol, and baicalein) treatment groups refers to (B, C, D), respectively, (**B**) control group, and (**C**) marked expression in positive brown tubular expression in Cis group. (**D**) The positive brown tubular expression slightly decreased in renal sections from the α-tocopherol group, (**E**) moderately decreased in renal sections from the baicalein group, and (**F**) significantly decreased in renal sections from the combined treatment group. Black arrows indicate a positive reaction. IHC counterstained with Mayer’s hematoxylin. High magnification ×: 400 bar 50.

The cisplatin group exhibited a significantly high TLR4 expression score when compared to the control group (*p* < 0.01). The score declined in α-tocopherol, baicalein, and combined treatment groups (Figure 8A). Figure 8B shows negative expression in kidney tissues from the control group, while kidney specimens of cisplatin group showed significant expression of TLR4 (Figure 8C). In contrast, the α-tocopherol group showed moderate expression (Figure 8D) and baicalein group showed mild expression (Figure 8E). TLR4 expression became significantly low in the combined treatment group (Figure 8F).

**Figure 9:**
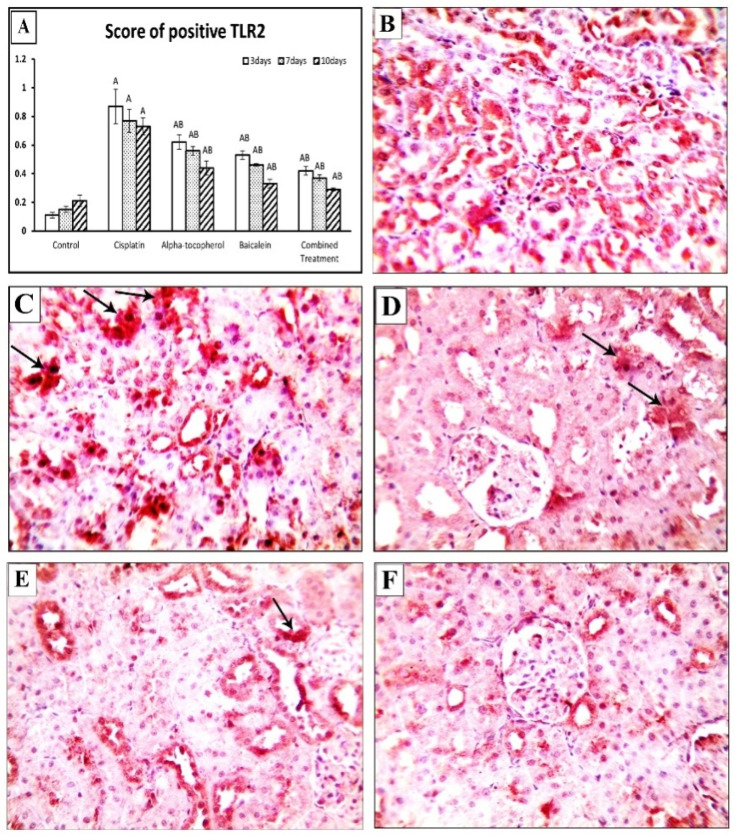
Microscopic pictures of adrenal sections immunostained against TLR2 from rat groups sacrificed after 10 days showing (**A**) positive cell score, significant vs. control group refers to A, while significant vs. (cisplatin, -tocopherol, and baicalein) treatment groups refers to (B, C, D), respectively, (**B**) control group, and (**C**) marked expression in positive brown tubular expression in Cisplatin group. (**D**) The positive brown tubular expression slightly decreased in renal sections from α-tocopherol group, (**E**) moderately decreased in renal sections from baicalein group, and (**F**) significantly decreased in renal sections from the combined treatment group. Black arrows indicate positive reaction. IHC counterstained with Mayer’s hematoxylin. High magnification ×: 400 bar 50.

Similarly, the cisplatin group showed a significantly high cytoplasmic expression of TLR2 (*p* < 0.01), which was attenuated by treatment with α-tocopherol, baicalein, and their combination (Figure 9A). Figure 9B shows negative expression in kidney tissues from the control group, while kidney samples of cisplatin group showed apparent cytoplasmic expression of TLR2 (Figure 9C). In contrast, the α-tocopherol group showed moderate expression (Figure 9D), and Baicalein group showed mild expression (Figure 9E). TLR2 expression became significantly low in the combined treatment group (Figure 9F).

**Figure 10:**
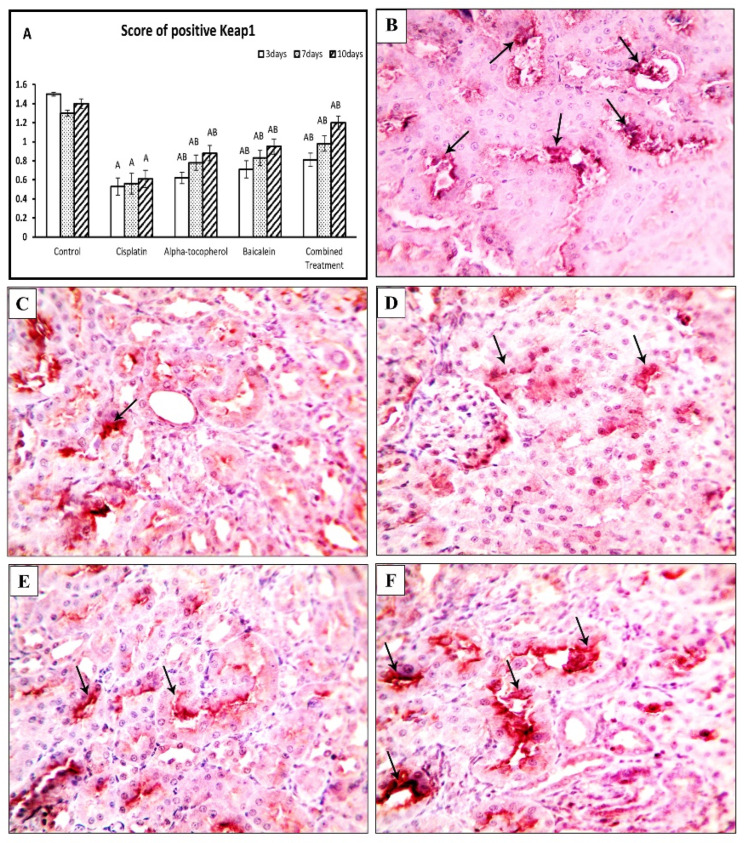
Microscopic pictures of adrenal sections immunostained against Keap-1 from rat groups sacrificed after 10 days showing (**A**) positive cell score, significant vs. control group refers to A, while significant vs. (cisplatin, -tocopherol, and baicalein) treatment groups refers to (B, C, D), respectively, (**B**) control group with marked Keap-1 expression, and (**C**) mild expression in positive brown tubular expression in Cisplatin group. (**D**) The positive brown tubular expression slightly increased in renal sections from the α-tocopherol group, (**E**) moderately increased in renal sections from the baicalein group, and (**F**) significantly increased in renal sections from the combined treatment group. Black arrows indicate positive reaction. IHC counterstained with Mayer’s hematoxylin. High magnification ×: 400 bar 50.

In contrast, the cisplatin group showed a decreased score of Keap-1 (*p* < 0.01), and this score became higher in α-tocopherol, baicalein, and combined treatment groups at different periods of time (Figure 10A). Figure 10B shows positive cells in kidney tissues from the control group, while kidney specimens obtained from the cisplatin group showed mild expression of Keap-1 (Figure 10C). On the other hand, the α-tocopherol group showed moderate expression (Figure 8D), and the baicalein group showed marked expression (Figure 10E). Keap-1 expression became significantly high in the combination group (Figure 10F).

## 3. Discussion

In 1978 and 1979, cisplatin was approved for medical use [19]. It is used to treat a wide range of cancers [20,21,22,23,24]. Unfortunately, it has a range of drawbacks that restrict its use, including nausea, vomiting, electrolyte disturbance, hemolytic anemia, and neurotoxicity [25]. Nephrotoxicity is a major concern. Until now, the nephrotoxicity mechanisms remain incompletely clear. In the present study, cisplatin administration increased BUN and creatinine levels, indicating reduced renal functions and hence nephrotoxicity, which has recently been associated with the stimulation of iNOS production that increases serum BUN and creatinine levels [26]. This reflects the histological alterations in the kidneys; it appears that baicalein and α-tocopherol have been protective, and the harmful effects of cisplatin are counteracted. This is in agreement with previously published studies that proved the renal protective role of vitamin E [27,28] and baicalein [29,30] as well. Furthermore, the combined therapy exhibited the most effective restoration of the renal function biomarkers. 

Cisplatin was identified to increase ROS by enhancing the oxidation of proteins, lipids, and nucleic acids [4]. The oxidative process and accumulation of ROS and renal cell injury through stimulation of apoptosis lead to cisplatin-induced nephrotoxicity [5]. In this study, cisplatin significantly decreased SOD and GSH, which reflects the severe side effects of cisplatin. Treatment with α-tocopherol, baicalein, and combined treatment exhibited a significant restoration in both SOD and GSH. This increase was seen gradually and was time-dependent, i.e., the intensity increased with the increase in the period of the treatment. This is consistent with other findings, which showed that in nephrotoxicity, there is a decrease in the efficiency of antioxidant enzymes such GPX, CAT, and SOD and antioxidant compounds such as glutathione, and there is an elevation in the lipid peroxidation levels [4]. The increased MDA content indicates lipid peroxidation, which is consistent with previous studies in which cisplatin administration resulted in inflammation and lipid peroxidation [31,32]. This may be attributable to a rise in the amount of released renal hydrogen peroxide because of the diminished antioxidant enzyme activity. The combined treatment of baicalein and α-tocopherol attenuated renal oxidative stress and restored the antioxidant balance to near normal.

Inflammation is a pathophysiological reaction to a wide range of disorders, including nephropathy, acute and chronic renal diseases, and kidney failure [33]. A vascular damage triggers an orderly process that increases the secretion of adhesion molecules, chemotactic factors, and cytokines. In this study, there was an elevation in NF-κB, IL-6, and TNF after administration of cisplatin. This reflects the inflammation induction effect of cisplatin. Previous studies reported that NF-κB–IL-6 signaling pathway has an important effect on vascular inflammation [34]. Earlier studies reported that there is increased renal expression of TNF in cisplatin-induced renal damage [35,36]. 

NF-κB responds to mediators of vascular injury leading to enhancing inflammatory genes, including adhesion molecules and chemotaxis [37]. This association between NF-κB and inflammatory diseases highlighted the importance of the development of therapeutic agents based on NF-κB inhibition [38]. Upon treatment with α-tocopherol, baicalein, and combined treatment, there was a decline in NF-κB, IL-6, and TNF in nephrotoxicity-induced groups. This, in turn, decreases the inflammatory response and would alleviate the serious side effects of cisplatin.

The interaction of TLRs and the immune system appears to be complicated in the context of AKI. TLRs are crucial in triggering immunological responses that stimulate cells and induce the release of proinflammatory cytokines, causing a greater immunoinflammatory response [39]. TLRs can function differently in AKI progression depending on their particular activation signal [40]. TLR2 and TLR4 represent innate immune receptors that can have different effects on kidney pathology. They are expressed in the collecting ducts, tubules, and Henle loop [41]. Our findings showed a significant upregulation of TLR2 and TLR4 expression levels in the cisplatin-treated group, which revealed the role of TLR2 and TLR4 in renal injury as it induces pathologic characteristics in AKI, including cisplatin-induced ones [42,43].

On the other hand, administration of α-tocopherol could reduce TLR2 and TLR4 expression, which agrees with the results of Tahan et al. [44], who hypothesized that vitamin E’s anti-inflammatory activity is due to a reduction in neutrophil function or cytokine production in the colon mucosa. Moreover, baicalein-treated groups showed a significant decrease in TLR4 gene and protein expression. This is consistent with the results of Lin, et al. [41], who revealed that baicalein administration reduces TLR2 and TLR4 stimulation and NF-κB signaling in kidneys after ischemia–reperfusion injury. Furthermore, coadministration of both α-tocopherol and baicalein could significantly reduce the expression of TLR4 and TLR2 compared to the administration of each alone.

Nrf2 is a primary transcriptional regulator of oxidative stress resistance [45]. In normal circumstances, the negative regulator Keap-1 (Kelch-like ECH-associated protein 1) keeps Nrf2 sequestered, but Keap-1 is inhibited under oxidative stress, and Nrf2 escapes degradation [46]. This theory agrees with our findings that showed a significant decline in the Keap-1 gene expression in the cisplatin group. In contrast, treatment with α-tocopherol could enhance the expression of Keap-1. That agrees with Feng, et al. [18], who reported the stimulation of the Keap-1/Nrf2 pathway by the induction of Nrf2 expression and the driving of its nuclear translocation after administration of α-tocopherol.

Moreover, the administration of baicalein showed more improvement in the gene and protein expression of Keap-1. That finding agrees with Sahu, et al. [9], who showed that baicalein treatment increased Nrf2 accumulation in the nucleus by modulating the Nrf2/Keap-1 pathway through a Keap-1-independent mechanism. Furthermore, administration of both α-tocopherol and baicalein showed the highest level of Keap-1, which confirms the antioxidant role of both against cisplatin-induced nephrotoxicity.

## 4. Materials and Methods

### 4.1. Experimental Animals

Male Sprague Dawley rats weighing 160–200 g (aged 2–3 months) were used. They were housed in separate cages provided with tap water and food. They were placed in a controlled environment, with room temperature maintained at 24 °C and 50–70% humidity. All experiments were conducted according to the NIH Guide for the Care and Use of Laboratory Animals and approved by “Mansoura Faculty of Medicine Institutional Research Board (MFM-IRB), Mansoura University, Egypt, under number R.20.11.1059”.

### 4.2. Study Design

Five rat groups with eighteen rat in each group were divided as follows: control group, rats were injected i.p. with 0.9% saline; cisplatin-treated group, rats were injected i.p. with cisplatin 3 mg/kg (Sigma-Aldrich, St. Louis, MO, USA);α-tocopherol group, animals received a single dose of i.p. α-tocopherol (250 mg/kg) 24 h prior to a single injection of cisplatin; baicalein group, rats received oral baicalein (100 mg/kg) for 7 consecutive days (Sigma-Aldrich, St. Louis, MO, USA) and a single i.p. injection of cisplatin on the fifth day; combined treatment group (cisplatin + α-tocopherol + baicalein group),rats received oral baicalein for 7 consecutive days and a single i.p. dose of α-tocopherol 24 h before a single injection of cisplatin on the fifth day.

Each group was then separated into three subgroups of six rats each, based on when they were sacrificed: 3 days, 7 days, and 10 days following cisplatin administration. Finally, rats were weighed, and blood was taken from the ocular venous plexus under inhalational general anesthesia. For creatinine and BUN measurements, blood samples were centrifuged at 220× *g* for 10 min, and serum was extracted and kept at 20 °C. Then, animals were sacrificed by cervical dislocation, a midline laparotomy was done, and both kidneys were removed. One kidney was placed in 10% buffered formalin for histopathological examination, and the other kidney was stored at −80°C for molecular studies.

### 4.3. Investigated Parameters

#### 4.3.1. Biochemical Assay

Measurements of serum creatinine and blood urea nitrogen (BUN) were done using commercially available kits according to manufacturer’s instructions using an autoanalyzer(CX 7; Beckman, Foster City, CA, USA).

#### 4.3.2. Evaluation of Oxidant/Antioxidant Parameters

The activity of superoxide dismutase (SOD) (Cat No. SD 25 21), levels of glutathione (GSH) (Cat No. TA 25 11), and malondialdehyde (MDA) (Cat No. MD 25 29) were tested in all study groups using commercial kits following the manufacturer’s instructions to determine the oxidative stress status in kidney tissues (Biodiagnostic Co., Giza, Egypt).

#### 4.3.3. Histopathological Studies

The kidney specimen was embedded in paraffin, and sections with 3 µm thickness were obtained and stained with hematoxylin and eosin and examined by light microscopy. Histopathological changes were analyzed in the different kidney regions (cortex and medulla) according to the scoring system [47]. Tubulointerstitial injury includes tubular necrosis and inflammatory cell infiltration of the interstitium. Regenerative changes include the presence of mitosis, solid cellular sheets between the tubules, intraluminal cellular proliferation with prominent hyperchromatic nuclei and little cytoplasm, giving the luminal border a festooned appearance.

#### 4.3.4. Immunohistochemistry

The expression levels of Keap-1, TLR2, and TLR4 were detected in the renal tissue of all studied groups using immunohistochemical staining. Briefly, paraffin-embedded sections were subjected to antigen retrieval citrate buffer for 30 min. Then, the expression levels of Kelch-like ECH associatedprotein1 (Keap-1) (cat:sc-514914, Santa Cruz), toll-like receptor 4 (TLR4) (cat: A5258, AB clonal), and toll-like receptor 2 (TLR2) (cat: A11225, AB clonal) were detected at 1:100 dilution; biotinylated secondary antibody was added after washes, and the slides washed again with PBS. Slides were incubated with substrate reagent diaminobenzidine (DAB) staining. The slides were counterstained with hematoxylin, dehydrated, mounted with a coverslip, and observed under an Olympus BX51 light microscope. The expression levels of TLR4, TLR2, and Keap-1 revealed brown nuclear staining, and the numbers of positive nuclei were calculated in ten nonoverlapping, randomly selected high-power fields of each slide [48].

#### 4.3.5. Gene Expression Assays for mRNA

Total RNAs from renal tissues were isolated using Triazol (Invitrogen). A Nano-Drop 2000c (Thermo Scientific, Germany was used to determine the concentration and purity of RNA samples). Following the manufacturer’s instructions, complementary DNA was generated from 1g/L RNA samples using the cDNA reverse transcription kit (Applied Biosystems, Foster City, CA, USA). QRT-PCR was performed using the primers of toll-like receptors and NF-KB pathway (NF-KB, IL-6, TNF, TLR2, TLR4, Nrf2, and Keap-1) shown in Table 1. Quantitative RT-PCR was performed as follows: initial denaturation at 95 °C for 10 min, 40 cycles at 95 °C for 15 s and 60 °C for 1 min, and an extension step at 72 °C for 1 min. The expression of studied genes and the housekeeping gene GAPDH was measured by using StepOnePlus real-time PCR (Applied Biosystems, Foster City, CA, USA) by the 2^−^^ΔΔCT^ equation.

#### 4.3.6. Statistical Analysis

The data were evaluated using one-way ANOVA, followed by post hoc Tukey’s test to compare the significant differences between various groups. Histopathological scores were described as nonparametric variables analyzed by Kruskal–Wallis test followed by Mann–Whitney test for all groups. A *p* value < 0.05 was considered statistically significant. (a vs. control, b vs. cisplatin, c vs. α-tocopherol, and d vs. baicalein). Results were expressed as mean ± standard error, and values of *p* ˂ 0.05 were regarded as statistically significantly different.

## 5. Conclusions

Cisplatin-induced higher plasma creatinine and urea levels were significantly reduced by baicalein, α-tocopherol, and combination therapy, which also counteracted cisplatin’s deleterious effects on oxidative stress markers and preserved tissues from cisplatin-induced lipid peroxidation. Furthermore, the combination of baicalein and α-tocopherol reduced the inflammatory response induced by cisplatin with a substantial decrease in NF-B, IL-6, TNF, TLR2, and TLR4 and a significant increase in Keap-1 and NRF-2. The combined therapy was the most successful and brought the condition close to normal status.

## Figures and Tables

**Table 1:** Primers used for quantitative RT-PCR.

Common Name	Sequence (5′-3′)	Accession Number
NF-KB	F: 5-GGACAGCACCACCTACGATG-3 R: 5-CTGGATCACTTCAATGGCCTC-3	NM_001276711.1
IL-6	F: 5-GCCCTTCAGGAACAGCTATGA-3 R: 5-TGTCAACAACATCAGTCCCAAGA-3	NM_012589.2
TNFα	F: 5-TTC GGA ACT CAC TGG ATC CC-3 R: 5-GGA ACA GTC TGG GAA GCT CT-3	NM_012675.3
TLR2	F: 5-TCCATGTCCTGGTTGACTGG-3 R: 5-AGGAGAAGGGCACAGCAGAC-3	NM_198769.2
TLR4	F: 5-GATTGCTCAGACATGGCAGTTTC-3 R: 5-CACTCGAGTAGGTGTTTCTGCTAA-3	NM_019178.2
Nrf2	F: 5-ATTGCTGTCCATCTCTGTCAG-3 R: 5-GCTATTTTCCATTCCCGAGTTAC-3	NM_001399173.1
Keap-1	F: 5-TGCTCAACCGCTTGCTGTATG-3 R: 5-CCAAGTGCTTCAGCAGGTACA-3	NM_057152.2
GAPDH	F: 5′-AGACAGCCGCATCTTCTTGT-3 R: 5′-TTCCCATTCTCAGCCTTGAC-3′	NM_017008.4

## Data Availability

All data that support the findings of this study are available from the corresponding author, upon request.

## Sample Availability

All Samples were used. Not Available at the moment.
